# Orally administered *Lactiplantibacillus plantarum* OLL2712 decreased intestinal permeability, especially in the ileum: Ingested lactic acid bacteria alleviated obesity-induced inflammation by collaborating with gut microbiota

**DOI:** 10.3389/fimmu.2023.1123052

**Published:** 2023-02-23

**Authors:** Yimei Wang, Tomohiro Takano, Yingyu Zhou, Rong Wang, Takayuki Toshimitsu, Toshihiro Sashihara, Masaru Tanokura, Takuya Miyakawa, Haruyo Nakajima-Adachi, Satoshi Hachimura

**Affiliations:** ^1^ Research Center for Food Safety, The University of Tokyo, Tokyo, Japan; ^2^ Department of Applied Biological Chemistry, The University of Tokyo, Tokyo, Japan; ^3^ Co-Creation Center, Meiji Holdings Co., Ltd, Hachiouji, Japan; ^4^ Graduate School of Biostudies, Kyoto University, Kyoto, Japan

**Keywords:** obesity, proinflammatory cytokines, macrophages, gut microbiota, intestinal permeability, lactic acid bacteria

## Abstract

**Introduction:**

Chronic inflammation caused by dietary obesity has been considered to induce lifestyle-related diseases and functional ingredients with anti-inflammatory effects are attracting attention. Although multiple studies on obesity had proved the anti-inflammatory effects of ingestion of lactic acid bacteria (LAB) and other functional ingredients on adipose tissue, the precise effects on the intestine, especially on the individual intestinal segments have not been made clear. In this study, we elucidated the mechanisms of *Lactiplantibacillus plantarum* (basonym: *Lactobacillus plantarum*) OLL2712 in suppressing obesity-induced inflammation using high fat diet (HFD)-fed mice obesity model.

**Methods:**

We orally administered heat-treated LAB to HFD-fed mice model, and investigated the inflammatory changes in adipose tissue and intestinal immune cells. We also analyzed gut microbiota, and evaluated the inflammation and permeability of the duodenum, jejunum, ileum and colon; four intestinal segments differing in gut bacteria composition and immune response.

**Results:**

After 3-week LAB administration, the gene expression levels of proinflammatory cytokines were downregulated in adipose tissue, colon, and Peyer’s patches (PP)-derived F4/80^+^ cells. The LAB treatment alleviated obesity-related gut microbiota imbalance. *L. plantarum* OLL2712 treatment helps maintain intestinal barrier function, especially in the ileum, possibly by preventing ZO-1 and Occludin downregulation.

**Discussion:**

Our results suggest that the oral administration of the LAB strain regulated the gut microbiota, suppressed intestinal inflammation, and improved the gut barrier, which could inhibit the products of obesity-induced gut dysbiosis from translocating into the bloodstream and the adipose tissue, through which the LAB finally alleviated the inflammation caused by dietary obesity. Barrier improvement was observed, especially in the ileum, suggesting collaborative modulation of the intestinal immune responses by ingested LAB and microbiota.

## 1 Introduction

According to the WHO Fact Sheet, worldwide obesity has nearly tripled since 1975, and the number of obese people is still rising due to the increased availability of high-calorie foods and lack of exercise, and it has become one of the most serious problems worldwide (1, 2). Multiple studies have shown that obesity can cause chronic inflammation (3–5). Persistent inflammatory conditions have been frequently reported to induce an exacerbation of lifestyle diseases, contributing to elevated risks of atherosclerosis, type 2 diabetes, and some cancers (6–8).

Gut microbiota, which represents the microorganisms in the gastrointestinal tracts of the animals, is mainly regulated by digested food. Gut microbiota is essential for the host metabolism, relating to the immune system and the barrier function (9, 10). Dysbiosis of gut microbiota is one of the key factors regulating obesity-associated disorders (11), as shown in the observation that germ-free mice do not show increased body fat mass or exacerbated insulin resistance when fed a high-fat (HFD) diet, and this phenomenon disappears after gut microbiota transplantation (12, 13). Multiple studies on gut microbiota in obese patients have suggested that obesity changes the gut microbiota, and excessive accumulation of adipose tissue is correlated with the composition of the gut microbiota. In addition, dietary obesity is known to reduce the diversity of the gut microbiota, followed by a disruption of the metabolic equilibrium, which is normally maintained by diverse components of the gut microbiota (14).

On the other hand, intestinal barrier dysfunction is also considered to be related to the aggravation of chronic inflammation caused by obesity. The gut is connected to the external environment for the absorption of nutrients. In the gastrointestinal tract, especially in the large intestine, there are large amounts of gut bacteria, as well as bacterial pathogens and other harmful substances. The intestinal barrier functions to protect the host from these harmful substances (15, 16). It has been reported that obesity increases intestinal permeability, which allows the leakage of inflammation inducible foreign substances such as lipopolysaccharide (LPS), which is one of cell component derived from Gram-negative bacteria. The leakage of Gram-negative bacteria and LPS into the bloodstream could induce the infiltration of proinflammatory macrophages in the adipose tissue and the liver tissue, inducing systemic inflammation (17). Furthermore, recent studies have suggested that dysbiosis results in intestinal inflammation in obesity (18).

Recently, functional ingredients with anti-inflammatory effects have received attention, from which lactic acid bacteria (LAB) are a diverse group of Gram-positive bacteria that produce lactic acid as the major end product of the carbohydrate fermentation, and are often considered as probiotics balancing the gut microbiota. As a LAB strain, *Lactiplantibacillus plantarum* OLL2712 has been selected owing to its capacity to accelerate the production of the anti-inflammatory cytokine interleukin (IL)-10 in murine marrow-derived dendritic cells (DCs) and peritoneal macrophages (19). Moreover, it has been reported that oral administration of *L. plantarum* OLL2712 alleviates chronic inflammation of adipose tissue in obese mouse models (20) and reduces fasting plasma glucose and serum proinflammatory cytokine concentrations in prediabetic individuals (21), suggesting that this functional LAB could be used as novel pharmaceuticals.

In this study, our main purpose was to focus on the intestine, especially on the different parts of the digestive tract. The anti-inflammatory functions of OLL2712 on the adipose tissue had been reported (19) but the pathways through which ingested OLL2712 exerted the anti-inflammatory effects on the adipose tissue remained unclear. In this regard, the effects on the intestine were unknown. We hypothesized that OLL2712 alleviated the adipocyte inflammation *via* the intestine by suppressing inflammation or enhancing the gut barrier, and we presumed that such functions were different among different parts. We investigated the mechanisms of the anti-inflammatory effects of the LAB strain, focusing on the gut microbiota and intestinal function using HFD-fed mouse model. We found a mechanism by which the oral administration of LAB regulated the gut microbiota, suppressed intestinal inflammation, and improved the gut barrier. This could inhibit bacterial harmful components, induced by obesity, from translocating into the bloodstream and adipose tissue, through which the LAB strain alleviated the inflammation caused by dietary obesity. Furthermore, the improvement of barrier function was observed, especially in the ileum of HFD-fed mice under the LAB treatment, suggesting collaborative modulation of the intestinal immune responses by the ingested LAB and microbiota.

## 2 Materials and methods

### 2.1 The LAB strain

*L. plantarum* OLL2712, which had been heat-treated by incubation at 75°C for 60 min and lyophilized, after being cultured in de Man, Rogosa, and Sharpe (MRS) broth (Becton Dickinson, USA), and stored at -20°C.

### 2.2 Mice and diet

C57BL/6 male mice were purchased from Charles River Laboratories (Japan, RRID : IMSR_CRL:027). Mice were fed sterilized (121°C, 20 min) water and maintained at an appropriate temperature (23 ± 2°C) and humidity (50 ± 5%) with a 12-hour light-dark cycle. All experiments were conducted with the approval of the Experimental Animal Ethics Committee of the Graduate School of Agriculture and Life Sciences of the University of Tokyo.

To create obese C57BL/6NCrl mice, mice were fed a HFD (60% kcal from fat; Oriental Yeast, Japan) from 8-week-old, and mice in the control group were fed a normal chow diet (AIN-93M; Oriental Yeast, Japan) (**Figure S1A**). 6 individuals were used for each group to investigate the proinflammatory changes induced by a 4-week HFD. The nutrient composition of HFD-60 and AIN-93M is shown in **Tables S1**, **S2**.

**Figure 1:**
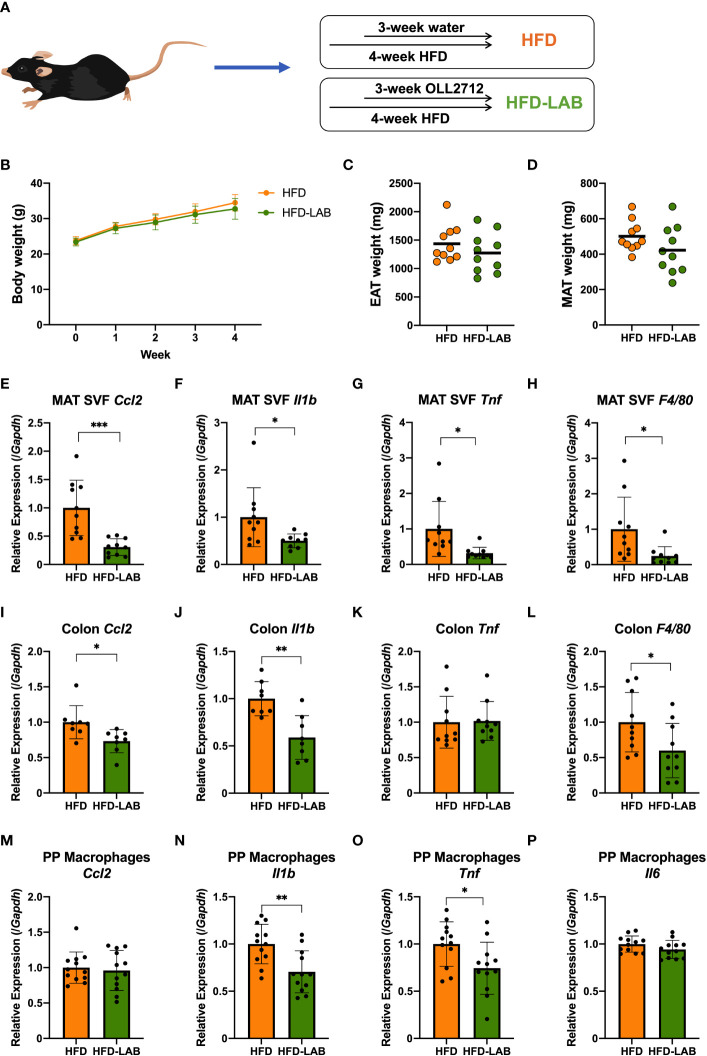
Oral administration of *L. plantarum* OLL2712 alleviated the inflammation of adipocytes, colon and PP macrophages in mice but changed neither the body weight nor the adipose tissue weight. C57BL/6N male mice were fed a HFD (60% kcal from fat) for 4 weeks from 8 weeks of age. **(A)** In the last 3 weeks, mice were administered OLL2712 daily (4 mg dissolved in 200 μL water per dose), and mice administered water simultaneously were used as a control group (HFD). **(B)** Mice were weighed once per week. The weights of the epididymal adipose tissue (EAT) **(C)** and mesenteric adipose tissue (MAT) **(D)** were measured after 3 weeks of treatment with OLL2712 and compared with the control group. The SVF cells were isolated from the MAT. The mRNA expression of CCL2 (*Ccl2*) **(E)**, IL-1β (*Il1b*) **(F)**, TNF (*Tnf*) **(G)**, and F4/80 (*F4/80*) **(H)** was measured by qPCR. The mRNA expression of CCL2 (*Ccl2*) **(I)**, IL-1β (*Il1b*) **(J)**, TNF (*Tnf*) **(K)**, and F4/80 (*F4/80*) **(L)** in colon tissue was measured by qPCR. Macrophages were isolated from PPs in the mice. The mRNA expression of CCL2 (*Ccl2*) **(M)**, IL-1β (*Il1b*) **(N)**, TNF (*Tnf*) **(O)**, and IL-6 (*Il6*) **(P)** was measured by qPCR. The results are representative of two independent experiments and are shown as the mean ± standard deviation (n = 8 - 12). *p<0.05; **p<0.01; ***p<0.001 (assessed using Student’s *t*-test). HFD, high-fat diet; LAB, lactic acid bacteria (*L. plantarum* OLL2712); MAT, mesenteric adipose tissue; SVF, stromal vascular fraction; PP, Peyer’s patch.

To investigate the effects of oral administration of *L. plantarum* OLL2712 in obese mice, C57BL/6NCrl mice were fed a HFD from 8-week-old for 4 weeks. In the last 3 weeks, *L. plantarum* OLL2712, suspended in the sterilized water to the concentration of 20 mg/mL, was orally administered every day, 4 mg to each mouse (**Figure 1A**). 8 – 12 individuals were used for each group.

To investigate the effects of a short-term oral administration of *L. plantarum* OLL2712 in mice, *L. plantarum* OLL2712, suspended in the sterilized water to the concentration of 20 mg/mL, was orally administered every day, 4 mg to each C57BL/6NCrl mouse from 9-week-old (**Figure S1B**). 5 individuals were used for each group.

### 2.3 Cell preparation

Epididymal adipose tissue (EAT) and mesenteric adipose tissue (MAT) were shredded into 2-3 mm pieces and dissociated with collagenase type II (1 mg/mL; Sigma-Aldrich, USA, Cat#C6885) at 37°C for 45-60 min until the adipose tissue was almost dissolved, and then the reaction was stopped with EDTA (10 mM) for 5 min. After being filtered with a 115 µm nylon mesh (Tokyo Screen, Japan), stromal vascular fraction (SVF) derived from adipose tissue was treated with red blood cell lysis buffer, made from ammonium chloride, potassium carbonate, and EDTA, for 5 min at room temperature. After centrifugation, the EAT SVF and MAT SVF were suspended in 10% FCS-RPMI.

Peyer’s patches (PPs) were treated with collagenase I (1 mg/mL; FUJIFILM Wako Pure Chemical, Japan, Cat#032-22364) and 10 μg/mL DNase I (Roche Diagnostics, Germany, Cat#10104159001) at 37°C for 60–90 min before being filtered with an 86 µm nylon mesh (Tokyo Screen, Japan). The PP cells were centrifuged twice and suspended in 10% FCS-RPMI. 10% FCS-RPMI was prepared using RPMI 1640 (Nissui Pharmaceutical, Japan, Cat#05918), containing 100 U/ml penicillin G potassium (Meiji Seika Pharma, Japan), 100 μg/ml streptomycin sulfate (Meiji Seika Pharma, Japan), 50 μM 2-mercaptoethanol (FUJIFILM Wako Pure Chemical, Japan, Cat#137-06862), 0.03% L-glutamine (FUJIFILM Wako Pure Chemical, Japan, Cat#074-00522), and 0.2% sodium hydrogen carbonate (FUJIFILM Wako Pure Chemical, Japan, Cat#191-01305), and 10% heat-inactivated fetal calf serum (Thermo Fisher Scientific, Germany, Cat#173012).

After F4/80 MicroBeads Ultrapure (Miltenyi Biotec, Germany, Cat#130-110-443) were added to PP whole cells, F4/80^+^ cells were isolated using a magnetic-activated cell sorting (MACS) system (Miltenyi Biotec, Germany). The obtained F4/80^+^ cells were used as macrophages.

### 2.4 Quantitative PCR

The intestinal contents were removed, and the intestinal tract was washed with PBS (-), added to TRIzol (Invitrogen, USA, 15596026), and homogenized using TissueRuptor (QIAGEN, Germany) until the tissue was barely visible. The intestinal tissue was immediately frozen in liquid nitrogen and stored at -80°C.

The intestinal tissue samples were thawed at 4°C. Then, 0.2 mL of chloroform (FUJIFILM Wako Pure Chemical, Japan, Cat#038-02606) was added to 1 mL of the sample in TRIzol reagent, and the mixture was stirred manually and kept at room temperature for 3 minutes. After centrifugation, the upper layer was transferred to a new tube, and 0.5 mL of isopropanol (FUJIFILM Wako Pure Chemical, Japan, Cat#166-04836) was added. After being kept at room temperature for 10 min, the sample was centrifuged. The sample was washed with 1 mL of 75% ethanol and dried at room temperature until the precipitate turned translucent. The RNA solution derived from the intestinal tissue was dissolved in sterilized water, and any DNA was removed using an RNase-Free DNase (QIAGEN, Cat#79254).

Total RNA from cells was isolated using QIAshredder (QIAGEN, Germany, Cat#79656), 2-mercaptoethanol (FUJIFILM Wako Pure Chemical, Japan, Cat#137-06862), and an RNeasy mini kit (QIAGEN, Cat#74106) according to the provided protocol. Complementary DNA (cDNA) was synthesized using SuperScript VILO MasterMix (ThermoFisher Scientific, USA, Cat#11755-050) and the GeneAmp PCR System 9700 (Applied Biosystems).

Synthesized cDNA samples were added to a LightCycler 480 Multiwell Plate 96 (Roche Diagnostics), and quantitative PCR was performed with a QuantiTect SYBR Green PCR Kit (QIAGEN, Cat#204143) using a CFX Connect Real-Time PCR Detection System (Bio-Rad, USA). The relative expression levels of each gene were standardized against the gene expression levels of glyceraldehyde-3-phosphate dehydrogenase (GAPDH). The primer sequences for quantitative PCR (qPCR) are shown in **Table S3**.

### 2.5 Measurement of intestinal permeability

Intestinal permeability *in vivo*, was measured using 4 kDa Fluorescein isothiocyanate-dextran (FITC-Dextran) (Sigma, Cat#46944). 4 hours after FITC-dextran was orally administered to mouse (12 mg per mouse), the serum was collected and diluted in a microplate reader (Greiner bio one, Austria), and the fluorescence was measured at 485(ex)/528(em) nm using a SpectraMax iD5 (Molecular Devices, Japan). The concentration of FITC-dextran was then calculated.

**Figure 2:**
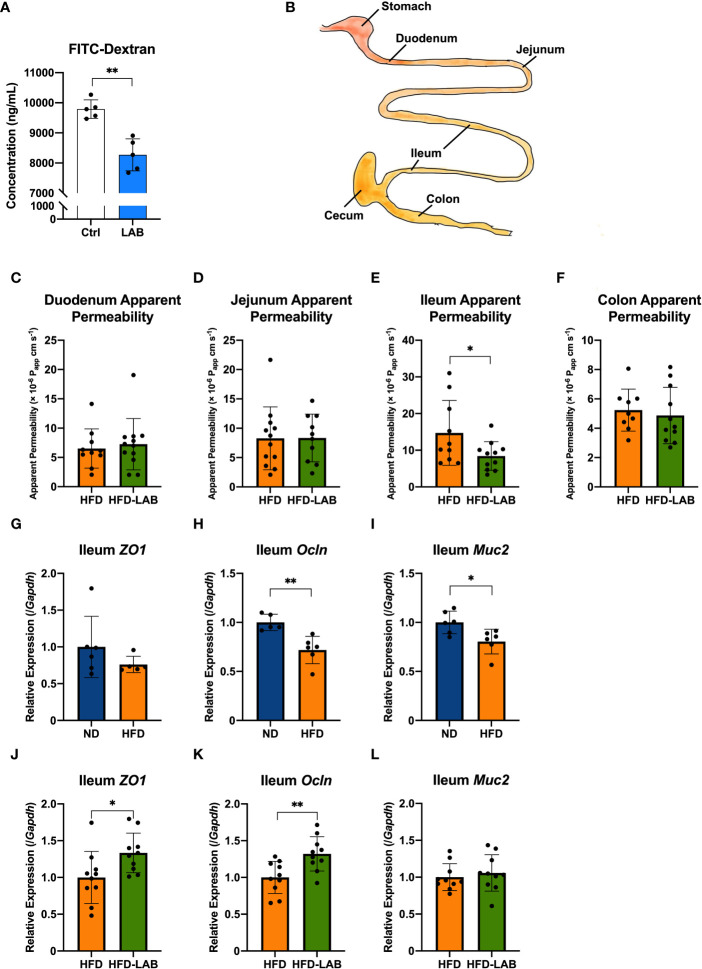
*L. plantarum* OLL2712 exhibited the ability to decrease intestinal permeability, especially in the distal intestine. **(A)** C57BL/6N male mice were treated with OLL2712 (4 mg dissolved in 200 μL water for each dose) for 7 days, and mice treated with water simultaneously were used as a control group (Ctrl) (n = 5). The concentration of FITC-dextran in serum was measured and calculated 4 hours after FITC-dextran was orally administered to mice to investigate epithelial permeability *in vivo*. **(B)** Specific intestinal sections were collected, and the permeability of each section was assessed. A 4 cm segment under the stomach was selected as the duodenum, a segment from the 5th to the 10th centimetre below the stomach as the jejunum, a 5 cm intestinal section proximal to the cecum as the ileum, and a 5 cm segment below the cecum as the colon. The apparent permeability of the duodenum **(C)**, jejunum **(D)**, ileum **(E)**, and colon **(F)** in C57BL/6N male mice fed an HFD and treated with OLL2712 (HFD-LAB) were compared with those fed an HFD and treated with sterilized water (HFD) (n = 9 - 12). The relative expression of ZO-1 (*ZO1*) **(G)**, Occludin (*Ocln*) **(H)**, and MUC2 **(I)** in ileal tissues from ND or HFD-fed mice was measured by qPCR (n = 5 - 6). The relative expression of ZO-1 (*ZO1*) **(J)**, Occludin (*Ocln*) **(K)**, and MUC2 **(L)** in ileal tissues from mice fed HFD and treated with OLL2712 (HFD-LAB) were compared with those fed HFD and treated with sterilized water (HFD) (n = 10). The results are representative of two independent experiments and are shown as the mean ± standard deviation. *p<0.05; **p<0.01 (assessed using Student’s *t*-test). Ctrl, control; LAB, lactic acid bacteria (*L. plantarum* OLL2712); ND, normal diet; HFD, high-fat diet.

Intestinal permeability *ex vivo* was investigated according to a previous report (22). The whole digestive tract from the stomach to the final part of colon was collected. After MAT was removed, specific intestinal sections were collected. A 4 cm segment under the stomach was selected as the duodenum, a segment from the 5th to the 10th centimetre below the stomach as the jejunum, a 5 cm intestinal section proximal to the cecum as the ileum, and a 5 cm segment below the cecum as the colon (**Figure 2B**). The collected intestinal tracts were washed by PBS (-) and the contents were gently removed without breaking the intestinal tissues. A 1 mg/mL solution of 4 kDa FITC-dextran was injected into the selected intestinal sections tied with surgical sutures, and each segment was moved to DMEM (Sigma-Aldrich, USA, Cat#11965092) and placed at 37°C (**Figure S1C**). The concentration of FITC-dextran transported from the lumen to the DMEM was measured every 30 minutes, and the cumulative concentration (Q_t_) of the DMEM, collected at each time point, was calculated using the following formula.

Q_t_ = (C_t_*V_r_) + (C_t sum_*V_s_), where:

Q_t_ = Cumulative concentration at time t

C_t_ = Concentration at time t (ng/mL)

V_r_ = Volume at the receiver side (mL)

C_tsum_ = Sum of all previous C_t_

V_s_ = Volume sampled (mL)

Q_t_ versus time (t) was plotted and the slope (δQ_t/_δt) was calculated. And the apparent permeability (P_app_) of each individual intestinal sac was calculated using the following formula:

P_app_ = (δQ_t/_δt)/(A*Co), where:

A = Area of tissue (cm^2^)

Co = Initial concentration (ng/mL)

### 2.6 Gut microbiota

The cecal contents were collected in 1.5 mL tubes and stored at -80°C. The gut microbiota was analysed with next-generation sequencing and amplicon sequencing by TechnoSuruga Laboratory (Japan). DNA was extracted according to the method previously reported (23), using an automated DNA isolation system (GENE PREP STAR PI-480 KURABO, Japan). The details of the analysis were provided by TechnoSuruga Laboratory (Japan). The 341f/R806 primers and dual-index method was used to amplify the V3-V4 regions of Bacterial 16S rRNA (23–26). And then barcoded amplicons were paired-end sequenced on 2×284-bp cycle using the MiSeq system with MiSeq Reagent Kit version 3 (600 Cycle) chemistry. Paired-end sequencing reads were merged by fastq-join ver 1.3.1 with default setting (27).

FASTX-Toolkit ver 0.0.13 was being used to extract joined-reads, which had quality value score of ≥ 20 for more than 99% of the sequence. After the chimeric sequences were deleted with usearch61 (28, 29), nonchimeric reads were submitted for 16S rDNA-based taxonomic analysis using the Ribosomal Database Project ver 2.11 (RDP, RRID : SCR_006633). Finally, Metagenome@KIN Ver 2.2.1 analysis software (World Fusion, Japan) was used to perform the identification with confidence ≥ 0.8.

### 2.7 Statistical analysis

The results are given as the mean ± SD, and Student’s *t*-test was used for statistical analyses. A difference was considered significant at *p*<0.05.

## 3 Results

### 3.1 Four-week intake of a high-fat diet caused inflammation in the SVF cells derived from adipose tissue in mice

**Figure 3:**
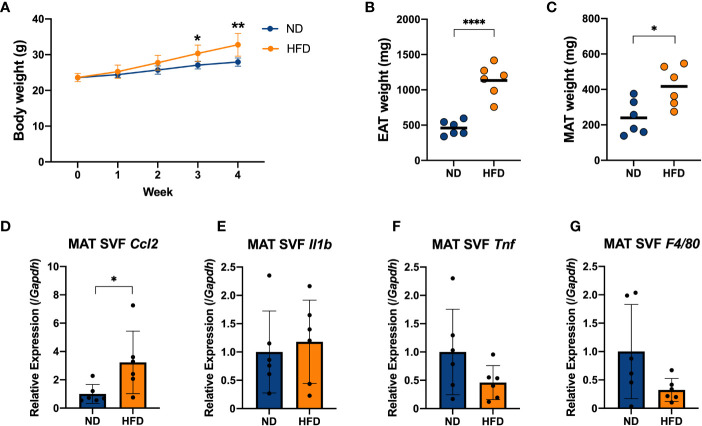
Four weeks of intake of a high-fat diet caused inflammation in the stromal vascular fraction (SVF) cells derived from adipose tissue of mice. C57BL/6N male mice were fed a HFD (60% kcal from fat) for 4 weeks from 8 weeks of age. Mice fed a standard chow diet (AIN-93 M) were used as a control group. **(A)** Mice were weighed once per week. The weights of the epididymal adipose tissue (EAT) **(B)** and mesenteric adipose tissue (MAT) **(C)** were measured after 4 weeks on a HFD and compared with the ND group. The stromal vascular fraction (SVF) cells were isolated from the MAT **(D–G)**. The mRNA expression of CCL2 (*Ccl2*), IL-1β (*Il1b*), TNF (*Tnf*) and F4/80 (*F4/80*) was measured by qPCR. The results are representative of two independent experiments and are shown as the mean ± standard deviation (n = 6). *p<0.05; **p<0.01; ****p<0.0001 (assessed using Student’s *t*-test). ND, normal diet; HFD, high-fat diet; EAT, epididymal adipose tissue; MAT, mesenteric adipose tissue; SVF, stromal vascular fraction.

It has been frequently reported that diet-induced obesity is related to chronic inflammation. A long period of intake of HFD, usually more than 12 weeks, could cause abnormal cytokine production, a disorder of lipid metabolism, and elevated blood glucose, followed by disruption of the regulatory mechanism of adipocytokine production (30). To investigate the inflammation in early obesity induced by a short period of HFD ingestion, C57BL/6N mice were fed HFD (60% kcal from fat) for 4 weeks, and mice fed a normal chow diet (AIN-93 M) were used as a control group (ND) (**Figure S1A**). The mice were weighed every 7 days, and it was found that the HFD group showed increasing body weights (**Figure 3A**), followed by increasing weight of their EAT and MAT (**Figures 3B, C**).

We isolated SVF, which contained immune cells, from the EAT and MAT, and the gene expression of proinflammatory cytokines in the EAT SVF and MAT SVF of mice was measured by qPCR (**Figures S2A–C**, **3D–F**). And the gene expression of F4/80 (*F4/80*), as a marker of macrophages, was measured simultaneously (**Figures S2D**, **3G**). Cytokine chemokine (C-C motif) ligand 2 (CCL2; *Ccl2*), as a macrophage-specific chemokine, increased with a 4-week HFD diet in EAT SVF and MAT SVF (**Figures S2A**, **3D**). Nevertheless, other major proinflammatory cytokine such as IL-1β (*Il1b*) and TNF (*Tnf*), and macrophage marker F4/80 (*F4/80*) did not show a remarkable change (**Figures S2B–D**, **3E–G**). These data suggested that a short-term of HFD feeding could induce increases in body weight and fat mass, followed by slight increase in inflammation in the adipose-derived SVF by inducing CCL2 (*Ccl2*).

### 3.2 Four-week intake of a HFD induced significant changes in the intestinal microbiota composition of mice

**Figure 4:**
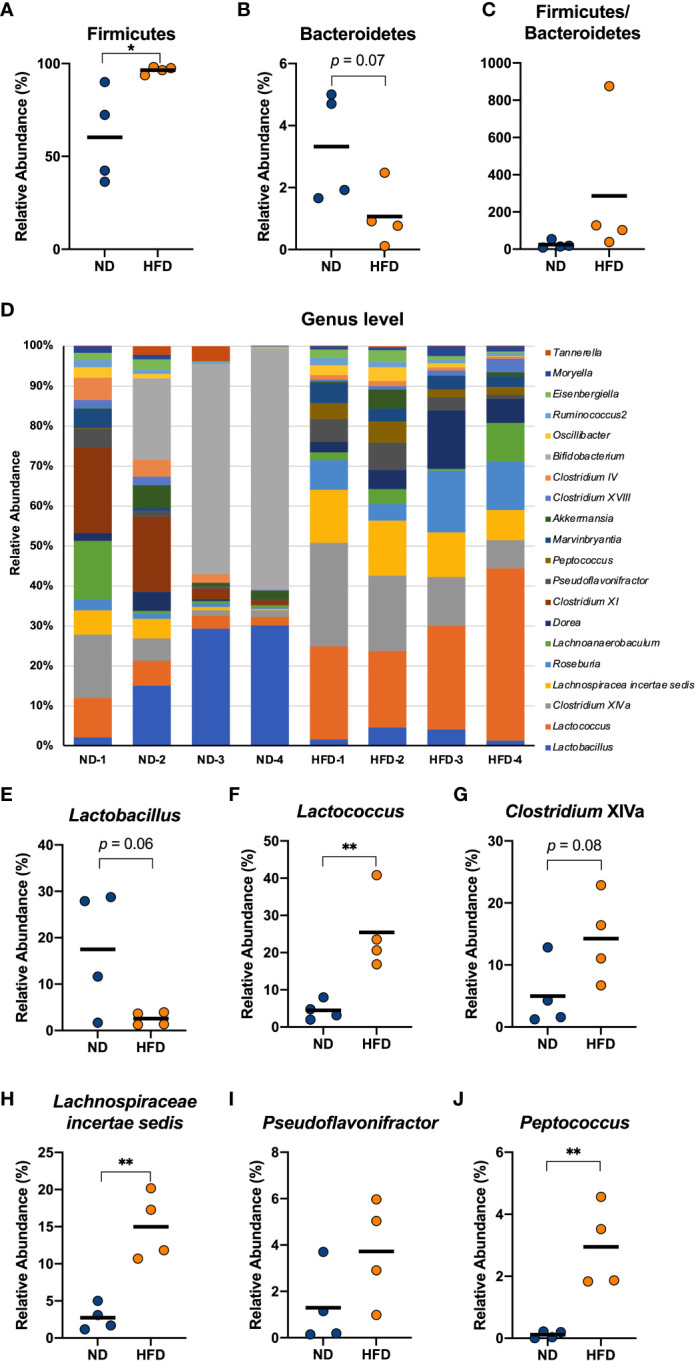
Four weeks of intake of a HFD caused significant changes in several genera derived from the cecum of mice. Male C57Bl/6N mice fed a HFD for 4 weeks were compared with mice fed a normal diet. The cecal contents of the mice were isolated, and the gut microbiota was investigated with next-generation sequencing applications. At the phylum level, the relative abundance of Firmicutes **(A)** and Bacteroidetes **(B)** and the ratio of the two **(C)** were calculated. At the genus level, the composition of the gut microbiota of each mouse was analysed and compared **(D)**. The relative abundance of *Lactobacillus* **(E)**, *Lactococcus* **(F)**, *Clostridium* XIVa **(G)**, *Lachnospiraceae incertae sedis* **(H)**, *Pseudoflavonifractor* **(I)**, and *Peptococcus* **(J)** in the HFD group were calculated and compared with those in the ND group. The results are representative of two independent experiments and are shown as the mean ± standard deviation (n = 6). *p<0.05; **p<0.01 (assessed using Student’s *t*-test). ND, normal diet; HFD, high-fat diet.

Many previous studies have reported that both obese patients and obese mice show an increase in Firmicutes and a decrease in Bacteroidetes in their gut microbiota (31, 32). We collected the contents of the cecum from mice fed a HFD for 4 weeks and analysed the gut microbiota composition using next-generation sequencing applications. The relative abundance of Firmicutes was found to increase in the HFD group compared to normal diet (ND) group (**Figure 4A**), which is a relevant marker of gut dysbiosis, and we detected a descending tendency in the relative abundance of Bacteroidetes (**Figure 4B**), although there was no change in the Firmicutes/Bacteroidetes ratio (**Figure 4C**). At the genus level, the mice in the HFD group showed an obvious distinction from the ND group (**Figure 4D**). The HFD group exhibited a decreasing tendency in the relative abundance of *Lactobacillus* (*p* = 0.06, **Figure 4E**) and the relative abundance of *Lactococcus*, *Lachnospiracea incertae sedis*, and *Peptococcus* which was almost absent in the cecum of the control group, was found to increase with a HFD feeding (**Figures 4F, H, J**). Although *Pseudoflavonifractor* did not change (**Figure 4I**), an increasing tendency induced by HFD was detected in the relative abundance of *Clostridium* cluster XIVa (*p* = 0.08, **Figure 4G**). These data indicated that a short-term HFD feeding had already resulted in a substantially different gut bacterial flora compared with the ND group, which could be involved in inflammation-associated diseases.

### 3.3 Oral administration of *L. plantarum* OLL2712 alleviated inflammation in SVF cells derived from adipose tissue

To explore the anti-inflammatory effects of *L. plantarum* OLL2712, we orally administered the heat-treated strain to mice on a HFD during the last 3 weeks (**Figure 1A**). We were unable to detect a significant difference in body weight or fat mass between mice treated with *L. plantarum* OLL2712 and those treated with sterilized water (**Figures 1B–D**). Nevertheless, in the EAT SVF, the gene expression of F4/80 (*F4/80*) decreased significantly in the LAB-treated mice (**Figure S3D**). Although not significant, the gene expression of CCL2 (*Ccl2*) showed a declining tendency with the LAB treatment (*p* = 0.05) (**Figure S3A**), while there was no change found in the gene expression of IL-1β (*Il1b*) and TNF (*Tnf*) (**Figures S3B, C**). Simultaneously, remarkable changes were found in the MAT SVF, as the gene expression of proinflammatory cytokines, CCL2 (*Ccl2*), IL-1β (*Il1b*) and TNF (*Tnf*), and macrophages marker, F4/80 (*F4/80*), decreased in the LAB group (**Figures 1E–H**).

### 3.4 Colon inflammation was suppressed by LAB treatment

Colonic macrophages play important roles in the induction of obesity-associated insulin resistance. Both macrophage-specific CCR2 knockout and intestinal epithelial cell-specific tamoxifen-inducible CCL2 knockout mice have been observed to be resistant to HFD, showing improved glucose and insulin tolerance (18). To investigate the colonic macrophage infiltration underlying dietary obesity, we evaluated the changes of CCL2 and F4/80 under HFD and LAB treatment. Unexpectedly, we did not find colonic inflammation in mice fed an HFD for 4 weeks (**Figures S4A–D**). Nevertheless, in macrophages derived from PP cells, the gene expression of CCL2 (*Ccl2*) and TNF (*Tnf*) was upregulated by dietary-induced obesity (**Figures S4E, G**), suggesting that intestinal inflammation was already elicited in the small intestinal compartment, although there was no detected change in the gene expression of IL-1β (*Il1b*) and IL-6 (*Il6*) in PP macrophages (**Figures S4F, H**).

On the other hand, a 3-week oral administration of *L. plantarum* OLL2712 elicited decreased expression of CCL2 (*Ccl2*), IL-1β (*Il1b*) and F4/80 (*F4/80*) (**Figures 1I, J, L**) in the colon, and TNF (*Tnf*) did not change in the gene expression levels (**Figure 1K**). Meanwhile, there was no change detected in the duodenum, jejunum, and ileum (data not shown). From these results, we supposed that the LAB strain had an anti-inflammatory effect on the large intestine of mice. Furthermore, with the oral administration of heat-treated OLL2712, the gene expression of the proinflammatory cytokines IL-1β (*Il1b*) and TNF (*Tnf*) were found to be downregulated in PP macrophages (**Figures 1N, O**), although CCL2 (*Ccl2*) and IL-6 (*Il6*) did not change (**Figures 1M, P**).

### 3.5 Gut microbiota bias caused by HFD intake was alleviated by an oral administration of *L. plantarum* OLL2712

**Figure 5:**
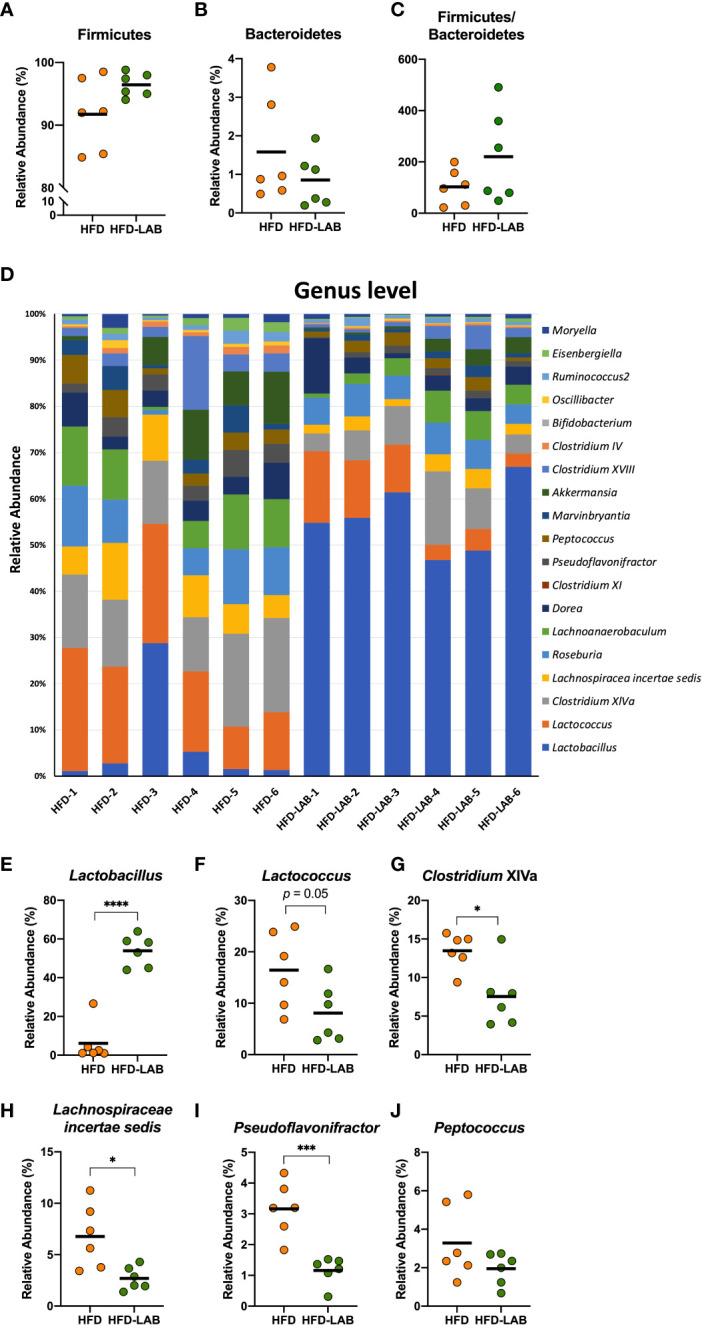
Oral administration of *L. plantarum* OLL2712 caused changes in the gut microbiota composition of mice. C57BL/6N male mice fed an HFD and treated with OLL2712 were compared with those fed an HFD and treated with sterilized water. The cecal contents of the mice were isolated, and the gut microbiota was investigated with next-generation sequencing applications. At the phylum level, the relative abundance of Firmicutes **(A)** and Bacteroidetes **(B)** and the ratio of the two **(C)** were calculated. At the genus level, the composition of the gut microbiota of each mouse was analysed and compared **(D)**. The relative abundance of *Lactobacillus* **(E)**, *Lactococcus* **(F)**, *Clostridium* XIVa **(G)**, *Lachnospiraceae incertae sedis* **(H)**, *Pseudoflavonifractor* **(I)**, and *Peptococcus* **(J)** in mice treated with OLL2712 were calculated and compared with those treated with water. The results are representative of two independent experiments and are shown as the mean ± standard deviation (n = 6). *p<0.05; ***p<0.001; ****p<0.0001 (assessed using Student’s *t*-test). HFD, high-fat diet; LAB, lactic acid bacteria (*L. plantarum* OLL2712).

We analysed the gut microbiota in the cecum of mice treated daily with heat-treated *L. plantarum* OLL2712 for 3 weeks compared with the control mice treated with water. At the phylum level, Firmicutes and Bacteroidetes did not show a significant change under the OLL2712 treatment (**Figures 5A–C**). However, at the genus level, the gut bacterial flora displayed a remarkable difference between mice treated with LAB and the control group treated with water (**Figure 5D**). The relative abundance of *Lactobacillus* dramatically increased (**Figure 5E**). Furthermore, the relative abundance of *Clostridium* cluster XIVa, *Lachnospiracea incertae sedis*, and *Pseudoflavonifractor*, which had been increased by the HFD and are considered to be associated with host inflammation and diseases, showed a significant decrease under OLL2712 treatment (**Figures 5G–I**). And the relative abundance of *Lactococcus* showed a declining tendency (*p* = 0.05) (**Figure 5F**), while *Peptococcus* did not change with the LAB treatment (**Figure 5J**). These data suggested that the oral administration of OLL2712 could modulate the gut microbiota composition related to obesity.

### 3.6 *L. plantarum* OLL2712 improved gut barrier function in the ileum

According to the experimental results obtained thus far, we confirmed the anti-inflammatory effects of heat-treated OLL2712 on adipose tissue, PP macrophages, and the colon. In addition, the LAB treatment caused a substantial change in the gut microbiome.

Obesity has been reported to increase intestinal permeability, after which the products of obesity-induced gut dysbiosis are allowed to translocate into the bloodstream, adipose tissue, or other organs, as one of the causes of chronic systemic inflammation (15, 33). Since intestinal inflammation and gut dysbiosis both affect the intestinal barrier (34–36), we next assessed the effects of the LAB strain on intestinal permeability. Seven days orally administration of heat-treated OLL2712 (**Figure S1B**) induced the lower levels of orally administered FITC-dextran in the serum compared to the control group, supporting our hypothesis (**Figure 2A**).

Since the proinflammatory cytokines in the colon decreased after 3 weeks of LAB treatment, while those in the jejunum did not, there was a possibility that different parts of the gut played different roles and might respond to the LAB strain in different ways. Assessment of the intestinal permeability in the previous *in vivo* experiment measured overall gastrointestinal absorption without any site specificity.

To further investigate site specificity of the protective effects of LAB treatment on the gut barrier, we collected duodenum, jejunum, ileum, and colon from the LAB treated mice and assessed the permeability *ex vivo* (**Figures 2B**, **S1C**). After 3 weeks of LAB treatment, the permeability of the ileum derived from HFD mice showed a significant reduction (**Figure 2E**), while the permeability of other segments had barely changed compared to that of HFD mice treated with water (**Figures 2C, D, F**). To gain mechanistic insight, we investigated the relative expression of barrier-related genes in each intestinal section. In the ileum, the expression of Occludin (*Ocln*), as one of the proteins forming tight junctions, decreased with a 4-week HFD feeding (**Figure 2H**). In addition, the gene expression of a secreted mucin with a physical barrier function, MUC2 (*Muc2*), also decreased with obesity (**Figure 2I**). And there was no significant change detected in the gene expression of ZO-1 (*ZO1*) (**Figure 2G**). The gene expression of ZO-1 (*ZO1*) and Occludin (*Ocln*) increased with the LAB treatment (**Figures 2J, K**), although no significant change was found in MUC2 (*Muc2*) (**Figure 2L**). These data suggested that LAB treatment blocked intestinal barrier disruption in the ileum of HFD-fed mice.

## 4 Discussion

This study clarified the mechanism by which oral administration of *L. plantarum* OLL2712 suppressed obesity-induced inflammation. Ingested OLL2712 might directly regulate the gut microbiota in the large intestine and reduce harmful substances, which are derived from obesity-induced gut dysbiosis and leak into the blood, eventually relieving adipocyte inflammation. Simultaneously, the LAB strain enhanced the intestinal barrier, especially in the ileum, suggesting collaborative modulation of intestinal immune responses by ingested LAB and microbiota. As a result of the enhancement of the gut barrier, the leakage of harmful substances into the bloodstream was reduced, which resulted in anti-inflammatory changes in the adipose tissue.

Obesity, which is usually caused by unhealthy eating habits, can induce chronic inflammation, leading to high risks of metabolic and immunological diseases (37, 38). The suppression and prevention of obesity-induced chronic inflammation by functional components have been frequently investigated (39–41). As functional ingredients, multiple LAB strains have been proven to be anti-inflammatory probiotics (42, 43), among which *L. plantarum* OLL2712 was focused on due to its good ability to highly induce the anti-inflammatory cytokine IL-10 (19). In recent studies, *L. plantarum* OLL2712 has been shown to hamper obesity-induced inflammation *in vivo*, reducing proinflammatory cytokines in murine adipose tissue (20) and human serum (21). In this study, we confirmed the anti-inflammatory effects of the LAB strain in the early period of obesity, focused on the regulatory effects of OLL2712 on the intestinal environment, and investigated the pathway by which this LAB strain exerted anti-inflammatory effects.

First, we fed mice a HFD for 4 weeks to examine the inflammatory responses triggered by early obesity. We found an increasing body weight and an enlarging fat mass in mice, with proinflammatory cytokines increasing in the adipose tissue-derived SVF, such as adipocyte immune cells. With the daily administration of the LAB strain for 3 weeks in the early period of obesity, although there was no alteration found in body weight or fat mass, the expression of macrophage-specific chemokine CCL2 (*Ccl2*) and proinflammatory cytokine IL-1β (*Il1b*) decreased, suggesting that the LAB could alleviate the macrophage infiltration and inflammation of adipose tissue caused by obesity.

We treated mice with the heat-treated *L. plantarum* OLL2712, because in previous studies, it had been found that the strain demonstrated strong anti-inflammatory effects on bone marrow-derived dendritic cells and peritoneal macrophages after being heat-treated to 75°C (19). We considered that the anti-inflammatory effects of the strain were stabilized by this heat treatment.

We believe that orally administered OLL2712 first reached the intestine and did not exert its effect directly on adipose tissue. It is well known that gut bacterial bias and disruption of the intestinal barrier contribute to chronic inflammation in obesity (33, 44–47). Furthermore, it has been suggested that intestinal inflammation precedes the inflammation in the adipose tissue (18). Therefore, we focused on gut microbiota and intestinal inflammation, examining the pathways of *L. plantarum* OLL2712 before it suppressed adipocyte inflammation.

We collected cecal contents and investigated the microbiota alterations caused by early obesity and the LAB treatment. *Lactobacillus* showed a significant decrease with HFD and an increase with the LAB treatment. We consider there was a possibility that the large increase in *Lactobacillus* was partly due to the administration of OLL2712 and simultaneously it was also possibly induced by the change of other strains belonging to *Lactobacillus* genus. Simultaneously, we detected a significant difference in gut microbiota between the short-term HFD group and the ND group, with an increasing trend in *Lactococcus*, *Clostridium* cluster XIVa, *Lachnospiracea incertae sedis*, and *Pseudoflavonifractor*. We found those genera changed oppositely in response to treatment with OLL2712. The genera we focused on have been reported to be involved in inflammation-associated diseases. An upregulation of bile acids production was detected in the intestines and feces of obese rodents, being related to the host inflammation, and was reported to be correlated to an increase in abundance of *Lactococcus* (48). It is known that diet-induced obesity induces the overproduction of *Clostridium* cluster XIVa (49), increasing the levels of deoxycholic acid, a gut bacterial metabolite that can cause DNA damage and is involved in the enhancement of obesity-associated hepatocellular carcinoma development in mice (50, 51). *Lachnospiracea incertae sedis* showed an enrichment in faecal samples of NAFLD (nonalcoholic fatty liver disease) patients (52), and *Pseudoflavonifractor* was reported to increase in the faeces of patients with ulcerative colitis (53). We cannot give a definite answer about whether OLL2712 was used as a food source by other bacteria or not. Nevertheless, there are multiple studies discussing that components derived from heat-sterilized products of LAB might feed intestinal bacteria and change the gut microbiota (54–56), which might be due to the proliferation of the gut bacteria that could easily utilize the active components of the LAB strain.

Furthermore, we examined intestinal inflammation by investigating the intestinal tissue in relation to their permeability as a hallmark of gut barrier enhancement (22). Considering that different parts of the gastrointestinal tract differ not only in their immune response but also in their number and composition of intestinal bacteria, we evaluated the inflammation and barrier function of the duodenum, jejunum, ileum, and colon to determine the effects of OLL2712 on each part of the intestine. We found that anti-inflammatory effects and intestinal barrier-enhancing effects of OLL2712 were exerted differently in the individual intestinal segments. The expression of CCL2 (*Ccl2*) and IL-1β (*Il1b*) was found to decrease in colon tissue but not in the small intestine after the LAB treatment. The apparent permeability of the ileum significantly decreased in response to the LAB treatment. Meanwhile, the gene expression of Occludin (*Ocln*) and MUC2 (*Muc2*) in ileum tissue declined in the HFD mice, while Occludin (*Ocln*) increased under the LAB treatment.

Occludin is well known as one of the proteins expressed in the intestine, forming tight junctions together with ZO-1 and claudins, which protect the body from harmful substances and pathogenic bacteria (57). MUC2 is a secreted mucin with a physical barrier function in the intestinal tract. Furthermore, we detected a significant decrease in serum FITC-dextran levels after 7-day treatment with LAB, which suggested that the administration of the LAB strain decreased the overall intestinal permeability (58). Therefore, it was suggested that OLL2712 could enhance the barrier function and alleviate adipocyte inflammation in obese mice by protecting them from harmful substances derived from the intestinal tract. Moreover, we found that such effects of *L. plantarum* OLL2712 on intestinal permeability were most noticeable in the ileum.

It was interesting that with the administration of *L. plantarum* OLL2712, the large intestine showed no change in barrier function, but the colonic inflammation was alleviated. Since there was a high possibility that the ingested LAB strain might not directly induce an immune response in the large intestine, our results suggested that the colonic inflammation might be alleviated by OLL2712 through regulating gut microbiota. On the other hand, there was no inflammatory change found in the small intestine tissue except for the PPs, but the barrier function was improved by the LAB strain in the distal part of the small intestine. The large intestine and small intestine are anatomically and functionally distinct (59). Most functions of the large intestine rely on gut bacteria (60), which include fermenting dietary fiber, producing SCFAs, and modulating the immune response (61, 62). On the other hand, the small intestine harbors lower numbers of commensal bacteria, such as segment filamentous bacteria, which mostly participate in the immune response by reacting directly to ingested food (63, 64). The duodenum is connected directly with the stomach, participating in food digestion (65), while the jejunum is believed to be involved in the immune response, as well as nutrient absorption (66). Compared to the jejunum, the ileum is closer to the large intestine, both physically and functionally. Meanwhile, unlike the large intestine, which cannot respond to orally ingested ingredients directly, in the ileum, multiple PPs are highly developed (67), and an immune response could be directly triggered by ingested food. Thus, the ileum, which is located in the final part of the small intestine, is the gut tract both easily influenced by gut microbiota and directly affected by the immunomodulatory effects of ingested components (68).

Therefore, we hypothesized that the administered *L. plantarum* OLL2712 might decrease intestinal permeability by cooperating with the gut microbiota *via* modulating the intestinal production of SCFAs. SCFAs, such as acetate and butyrate produced by the balanced bacteria, could inhibit the pathways of hyodeoxycholic acid (HDCA) or NF-κB to alleviate the intestinal inflammation and enhance the gut barrier (69). On the other hand, OLL2712 might also enhance the barrier by inducing the production of IgA, which may protect the intestinal epithelial cells from LPS and pathogenic bacteria and alleviated the intestinal inflammation (70). Nevertheless, we consider there was still a possibility that OLL2712 function directly on the intestinal epithelial cells *via* Toll-like receptors (especially TLR2) or Nod-like receptors, well known as the pathways through which the intestinal epithelial cells recognized the bacteria (71–73). Further studies especially *in vitro* experiments to co-culture SCFAs or the intestinal contents and OLL2712, are needed to confirm the hypothesis.

**Figure 6:**
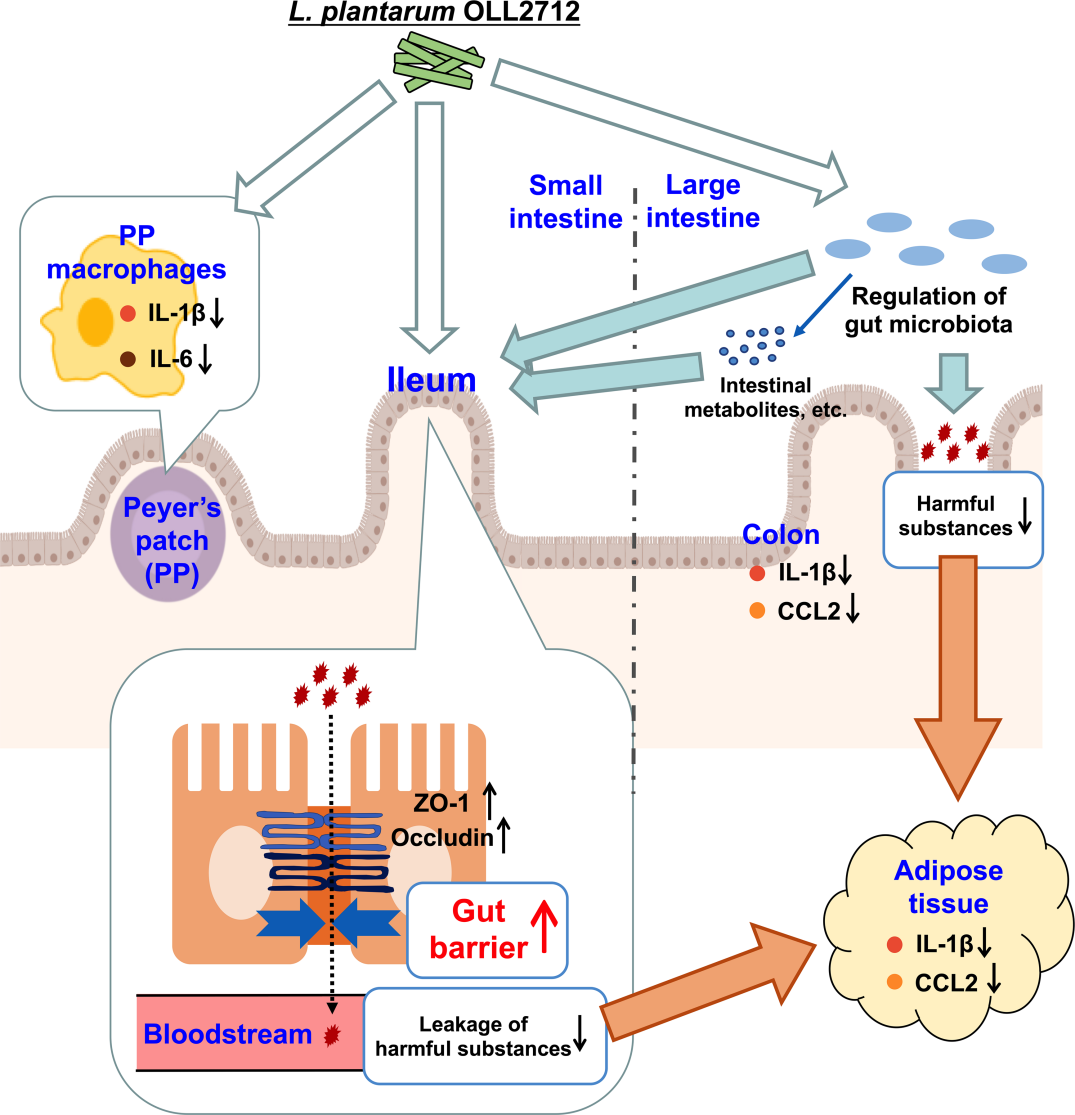
The pathways by which the lactic acid bacteria (LAB) strain exerted its anti-inflammatory effects. Ingested OLL2712 might directly regulate the gut microbiota in the large intestine and reduce harmful substances, which are derived from obesity-induced gut dysbiosis and leak into the blood, eventually relieving adipocyte inflammation. Simultaneously, the LAB strain enhanced the intestinal barrier, especially in the ileum, suggesting collaborative modulation of intestinal immune responses by ingested lactic acid bacteria and microbiota. The enhancement of the gut barrier reduced the leakage of harmful substances into the bloodstream, which resulted in anti-inflammatory changes in the adipose tissue.

The results of this research suggested that OLL2712 reached the small intestine, alleviating inflammation and cooperating with the gut bacteria to enhance barrier function, especially in the ileum. This prevented the leakage of harmful substances, thereby suppressing adipocyte inflammation (**Figure 6**). If intestinal substances that cooperate with OLL2712 and participate in anti-inflammatory effects can be identified, we could elucidate the mechanisms of the health function of LAB to alleviate metabolic diseases and chronic inflammation.

## Data availability statement

The data supporting this study are available from the corresponding author upon request.

## Ethics statement

The animal study was reviewed and approved by the Experimental Animal Ethics Committee of the Graduate School of Agriculture and Life Sciences of the University of Tokyo.

## Author contributions

YW and SH conceived this study. YW designed the research studies. YW, TTa, YZ and RW performed the experiments. YW analyzed the data and wrote the manuscript. TTo and TS prepared materials and reviewed the manuscript. TTa, YZ, RW, HN-A, TM, MT and SH reviewed the manuscript. All authors contributed to the article and approved the submitted version.
