# Supplementary material for: Orally administered Lactiplantibacillus plantarum OLL2712 decreased intestinal permeability, especially in the ileum: Ingested lactic acid bacteria alleviated obesity-induced inflammation by collaborating with gut microbiota
Source: Front Immunol. 2023 Feb 23;14:1123052. doi: 10.3389/fimmu.2023.1123052 (PMC9995389; doi:10.3389/fimmu.2023.1123052)
Supplement: Supplementary file 1 [file DataSheet_1.pdf]

Supplementary Material

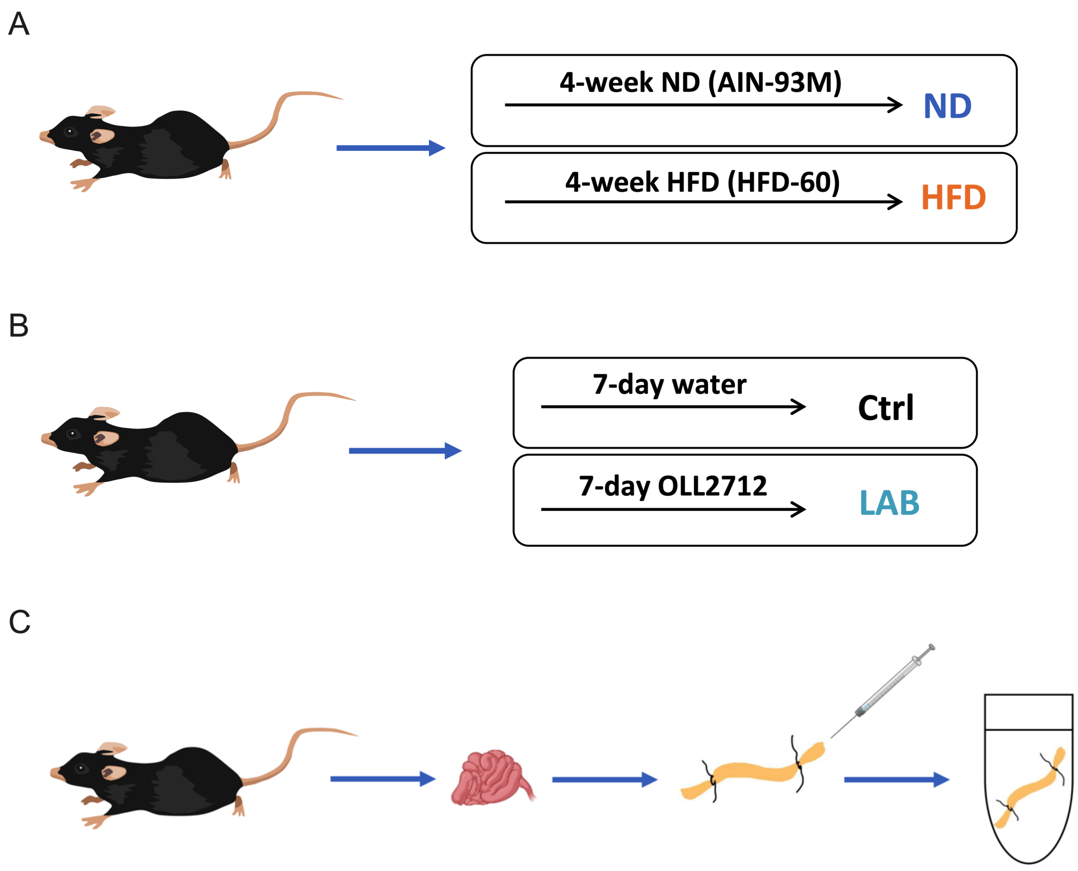

Figure S1. Experimental protocols were shown to investigate the effects of *L. plantarum* OLL2712. (A) C57BL/6N male mice were fed a HFD (60% kcal from fat) for 4 weeks from 8 weeks of age. Mice fed a normal diet (AIN-93 M) were used as a control group (ND). (B) *L. plantarum* OLL2712, suspended in the sterilized water to the concentration of 20 mg/mL, was orally administered every day, 4 mg to each C57BL/6NCrl mouse from 9-week-old. (C) A 1 mg/mL solution of 4kDa FITC-dextran was injected into the selected intestinal sections tied with surgical sutures, and each segment was moved to DMEM and placed at 37 °C. The concentration of FITC-dextran transported from the lumen to the DMEM was measured every 30 minutes. ND, normal diet; HFD, high-fat diet; Ctrl, control; LAB, lactic acid bacteria (*L. plantarum* OLL2712).

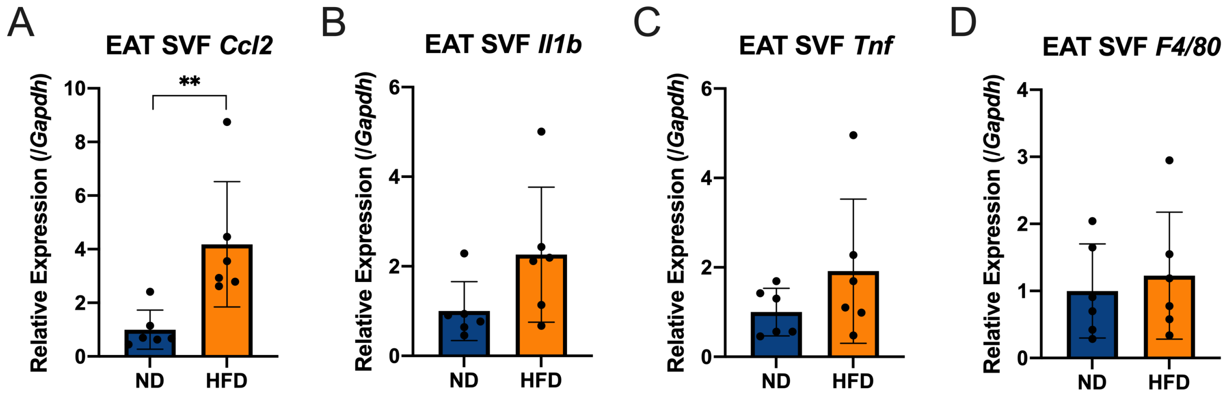

Figure S2. Four weeks of intake of a HFD caused inflammation in the SVF cells derived from EAT in mice. C57BL/6N male mice were fed a HFD (60% kcal from fat) for 4 weeks from 8 weeks of age. Mice fed a normal diet (AIN-93 M) were used as a control group. The SVF cells were isolated from EAT. The mRNA expression of CCL2 (*Ccl2*) (A), IL-1β (*Il1b*) (B), TNF (*Tnf*) (C), F4/80 (*F4/80*) (D) was measured by quantitative PCR (qPCR). The results are shown as the mean±standard deviation (n = 6). $^{**}p<0.01$ (assessed using Student's *t*-test). ND, normal diet; HFD, high-fat diet; EAT, epididymal adipose tissue; MAT, mesenteric adipose tissue; SVF, stromal vascular fraction.

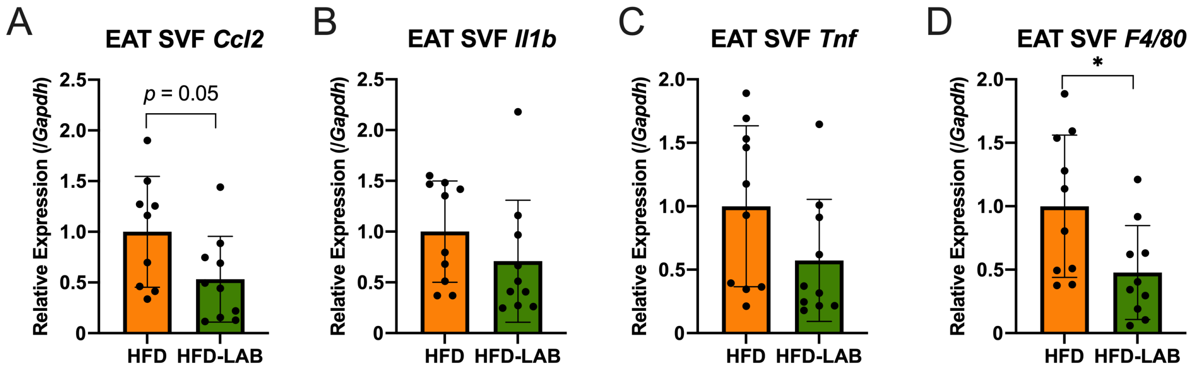

Figure S3. Oral administration of *L. plantarum* OLL2712 alleviated inflammation in SVF cells derived from EAT in mice. C57BL/6N male mice fed an HFD and treated with OLL2712 were compared with those fed an HFD and treated with sterilized water. The SVF cells were isolated from epididymal adipose tissue (EAT). The mRNA expression of CCL2 (*Ccl2*) (A), IL-1β (*Il1b*) (B), TNF (*Tnf*) (C), F4/80 (*F4/80*) (D) was measured by quantitative PCR (qPCR). The results are shown as the mean±standard error of the mean (n = 9 - 10). $^{*}p<0.05$ (assessed using Student's t-test). HFD, high-fat diet; Lac, *L. plantarum* OLL2712; EAT, epididymal adipose tissue; SVF, stromal vascular fraction.

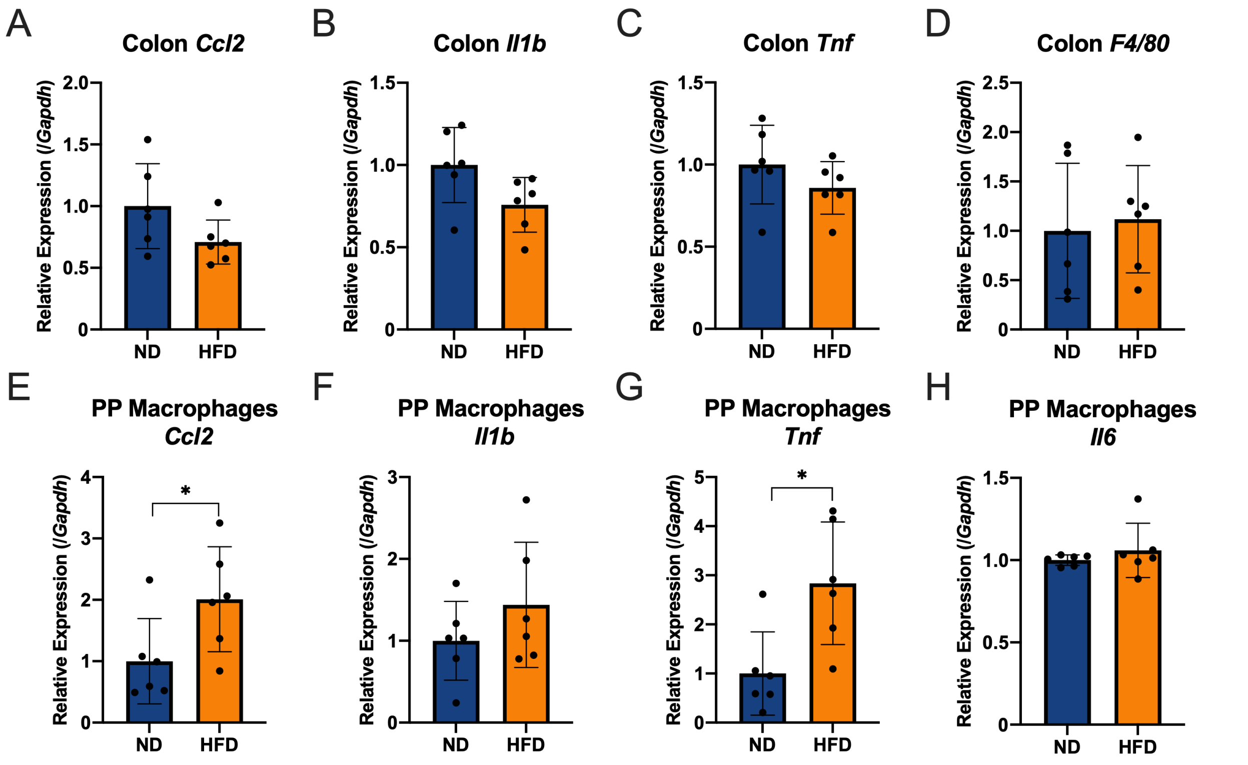

Figure S4. Proinflammatory changes were not observed in colon tissue but detected in PP macrophages derived from mice fed a high-fat diet for 4 weeks. Male C57Bl/6N mice fed a high-fat diet for 4 weeks were compared with mice fed a normal diet. The mRNA expression of CCL2 (*Ccl2*) (A), IL-1β (*Il1b*) (B), TNF (*Tnf*) (C), F4/80 (*F4/80*) (D) in colon tissue was measured by quantitative PCR (qPCR). Macrophages were isolated from Peyer's patches (PPs) in mice. The mRNA expression of CCL2 (*Ccl2*) (E), IL-1β (*Il1b*) (F), TNF (*Tnf*) (G), and IL-6 (*Il6*) (H) was measured by quantitative PCR (qPCR). The results are representative of two independent experiments and are shown as the mean±standard error of the mean (n = 6). *$p<0.05$ (assessed using Student's t-test). ND, normal diet; HFD, high-fat diet; PP, Peyer's patch.

Table S1 Composition of HFD (60 kcal % fat)

**Ingredient**	%
Casein	25.6
L-Cysteine	0.36
Maltodextrin	6.0
Pregelatinized corn starch	16.0
Sucrose	5.5
Soybean oil	2.0
Lard	33.0
Cellulose	6.6
Mineral mixture	3.5
Calcium carbonate	0.18
Vitamin mixture	1.0
Choline bitartrate	0.25
Total	100.0

Table S2 Composition of a normal diet (AIN-93M)

Ingredient	%
Casein	14.0
L-Cysteine	0.18
Corn starch	45.6
Pregelatinized corn starch	15.5
Sucrose	10.0
Soybean oil	4.0
Cellulose	5.0
Mineral mixture	3.5
Vitamin mixture	1.0
Choline bitartrate	0.25
tert-Butylhydroquinone	8×10^{-4}
Total	100

Table S3 Primer sequences for quantitative PCR

Targets	qPCR primers
GAPDH (*Gapdh*)	5' - TGT CCG TCG TGG ATC TGA C -3' (forward)
	5' - CCT GCT TCA CCA CCT TCT TG - 3' (reverse)
CCL2 (*Ccl2*)	5' - GGC TCA GCC AGA TGC AGT TAA C - 3' (forward)
	5' - GCC TAC TCA TTG GGA TCA TCT TG - 3' (reverse)
F4/80 (*F4/80*)	5' - TTT TCA GAT CCT TGG CCA TC - 3' (forward)
	5' - ACA CTG GGG CAC TTT TGT TC - 3' (reverse)
IL-1β (*Il1b*)	5' - CAG GAT GAG GAC ATG AGC AC - 3' (forward)
	5' - CAG TTG TCT AAT GGG AAC GTC A - 3' (reverse)
TNF (*Tnf*)	5' - CCA CCA CGC TCT TCT GTC TA - 3' (forward)
	5' - TGG GCT ACA GGC TTG TCA - 3' (reverse)
IL-6 (*Il6*)	5' - TGG AGT CAC AGA AGG AGT GGC TAA G - 3' (forward)
	5' - TCT GAC CAC AGT GAG GAA TGT CAA - 3' (reverse)
ZO-1 (*ZO1*)	5' - AAA TCA TCC GAC TCC TCG TC - 3' (forward)
	5' - CAG TTG GCT CCA ACA AGG TAA - 3' (reverse)
Occludin (*Ocln*)	5' - TGT GTG TGA GCT GTG ATG TGA - 3' (forward)
	5' - GGC TGC TGC AAA GAT TGA TT - 3' (reverse)
MUC2 (*Muc2)*	5' - ATG CCC ACC TCC TCA AAG AC - 3' (forward)
	5' - GTA GTT TCC GTT GGA ACA GTG AA - 3' (reverse)
